# Using Artificial Intelligence to Address Mental Health Inequalities in Low-Income, Urban Youth in North West England: A Digital Health Promotion Intervention

**DOI:** 10.1192/bjo.2025.10237

**Published:** 2025-06-20

**Authors:** Andi Stanescu

**Affiliations:** Manchester University NHS Foundation Trust, University of Manchester, Manchester, United Kingdom

## Abstract

**Aims:** This study aims to design and implement a digital health promotion intervention aimed at reducing mental health inequalities among low-income, urban youth in North West England. The intervention is grounded in the hypothesis that a combined approach – incorporating peer mentorship, digital technology, and community-driven initiatives – will enhance mental health awareness, reduce stigma, and increase engagement with mental health services in this vulnerable population.

**Methods:** The intervention consists of three key components: (1) Training 50 peer mentors to deliver mental health workshops in local schools, (2) Developing a culturally relevant digital mental health app that offers self-help tools and anonymous counselling, and (3) Hosting five community-based mental health awareness events to engage families and local leaders. The intervention is evaluated using a mixed-methods approach. A sample of 500 students will complete pre- and post-intervention surveys to assess changes in mental health literacy, stigma, and help-seeking behaviours. Focus groups will capture qualitative insights into participant experiences, while app analytics will track usage patterns, such as downloads, active users, and interaction with features. School attendance records will also be reviewed to assess the potential impact on student well-being. The evaluation will provide both quantitative and qualitative data to determine the intervention’s effectiveness and acceptability.

**Results:** The intervention is expected to significantly increase mental health awareness and literacy, with an anticipated 20% reduction in self-reported symptoms of anxiety and depression. The app is projected to achieve 1,000 downloads and 300 active users within the first 18 months of implementation. The peer mentorship programme is expected to foster a supportive environment in schools, helping to reduce mental health stigma and encouraging students to engage with available services. Additionally, the community-based events are predicted to engage over 300 young people and their families, further reducing stigma and promoting open conversations about mental health.

**Conclusion:** This intervention has the potential to significantly improve mental health outcomes for low-income, urban youth by addressing both systemic and individual barriers. The predicted results suggest that the model is feasible, scalable, and adaptable to similar socio-economic contexts. The next steps involve expanding the intervention to additional regions, enhancing collaboration with key stakeholders, and refining the digital components of the intervention based on user feedback. Acceptance of this study at this conference would offer an opportunity to share insights into community-driven approaches to tackling mental health inequalities and enhancing access to mental health resources.

